# A new genus and species of thorny lacewing from Upper Cretaceous Kuji amber, northeastern Japan (Neuroptera, Rhachiberothidae)

**DOI:** 10.3897/zookeys.802.28754

**Published:** 2018-12-04

**Authors:** Hiroshi Nakamine, Shûhei amamoto

**Affiliations:** 1 Minoh Park Insect Museum, Minoh Park 1-18, Minoh City, Osaka, 562-0002, Japan Minoh Park Insect Museum Minoh Japan; 2 Integrative Research Center, Field Museum of Natural History, 1400 S Lake Shore Drive, Chicago, IL 60605, USA Field Museum of Natural History Chicago United States of America

**Keywords:** fossil, Japan, Mantispoidea, Paraberothinae, Rhachiberothidae, Santonian

## Abstract

*Kujiberothateruyukii***gen. et sp. n.**, a remarkable new genus and species of Rhachiberothidae, is described from Upper Cretaceous amber from the Kuji area in northeastern Japan. This discovery represents the first record of this family both from Japan and from East Asia. This fossil taxon has the largest foreleg in the subfamily Paraberothinae found to date and its discovery implies that this group had higher morphological diversity in the Cretaceous than it does now. This finding also stresses the importance of the insect inclusions in Kuji amber, which have not been well explored in spite of their potential abundance.

## Introduction

Rhachiberothidae, or thorny lacewings, are a small family of Neuroptera, which have 13 extant species assigned to three genera as well as rather abundant fossil records and extinct taxa (Table 1): *Hoelzeliella* Aspöck & Aspöck, 1997, *Mucroberotha* Tjeder, 1959, and *Rhachiberotha* Tjeder, 1959 (Aspöck and Mansell 1994; [Bibr B3][Bibr B19]; Makarkin 2015a; Oswald 2018). This family has sometimes been treated as a subfamily (Rhachiberothinae) of Berothidae (e.g., Winterton et al. 2010; Makarkin 2015a), but here we tentatively follow the familial status of Rhachiberothidae on the basis of recent extensive studies (Winterton et al. 2018; Engel et al. 2018). The distribution of the extant rhachiberothids is restricted to sub-Saharan Africa with records from Ethiopia, Zimbabwe, Angola, Namibia, and South Africa (Aspöck and Aspöck 1997). Rhachiberothidae is known as a sister taxon to Berothidae (Aspöck and Mansell 1994; Aspöck et al. 2001, 2012) or Mantispidae (Liu et al. 2015; Engel et al. 2018). These families and the extinct family Mesoberothidae constitute the superfamily Mantispoidea (Winterton et al. 2018; Engel et al. 2018). Mesoberothidae was established by Riek (1955) based on the two forewing fossils from the Upper Triassic Mount Crosby Formation in Australia. This extinct family is considered to be a stem group of Berothidae or it even forms a sister group to the rest of Mantispoidea (Engel et al. 2018).

Rhachiberothidae comprises two subfamilies, Rhachiberothinae and Paraberothinae. Rhachiberothinae includes 13 extant species and two extinct species from mid-Eocene Baltic amber (Whalley 1983; Engel 2004; Makarkin and Kupryjanowicz 2010). Paraberothinae is a uniformly extinct group, which occurred only in the Cretaceous. To date, it is composed of 13 valid species in 12 extinct genera, as well as a single undescribed species of uncertain generic placement. The subfamily is characterized with a combination of eleven morphological characters, e.g., small body size (forewing 2.9–4.2 mm long); antennal scapus long to very long; forelegs raptorial; at least two spines present on the inner edge of protibia (synapomorphy); ScP and RA fused distally in both fore- and hindwings; loss of the intermediate subcostal crossvein in the distal part of the forewing; CuP present in the hindwing (Nel et al. 2005a; Makarkin and Kupryjanowicz 2010; Makarkin 2015a). This group is known from various Cretaceous amber deposits, namely Burmese, Canadian, French, Lebanese and New Jersey amber (Schlüter 1978; Whalley 1980; Grimaldi 2000; Engel 2004; Nel et al. 2005a; Engel and Grimaldi 2008; McKellar and Engel 2009; Petrulevičius et al. 2010; Shi et al. 2015; Makarkin 2015a; Table 1). The taxonomic position of the monotypic species *Oiseacelinea* (Nel et al. 2005) (Nel et al. 2005a, b) from the earliest Eocene Oise amber remains uncertain within Rhachiberothidae (Makarkin and Kupryjanowicz 2010). There is no rhachiberothid compression fossil known from anywhere in the world, possibly because of their small, fragile bodies (Petrulevičius et al. 2010).

Fossil rhachiberothid has never been found from Japan or anywhere else in East Asia. Recently, we examined a rhachiberothid fossil, previously considered as a member of Mantodea (Delclòs et al. 2016), found in Upper Cretaceous amber (Santonian) from the Kuji area of northeastern Japan. Herein, a remarkable new genus and species of Paraberothinae is described based on this specimen. Our finding indicates that this subfamily was also distributed in the eastern part of Laurasia, further reinforcing the idea that the distribution of Paraberothinae was widespread. This discovery also suggests a higher morphological diversity of thorny lacewings than previously documented.

**Table 1.** List of the fossil Rhachiberothidae of the world.

Taxon	Deposit	Reference
** Paraberothinae **		
*Chimerhachiberothaacrasarii* Nel et al., 2005	Neocomian, Lebanese amber	Nel et al. 2005a
*Paraberothaacra* Whalley, 1980	Neocomian, Lebanese amber	Whalley 1980; Nel et al. 2005a
*Raptorapaxterribilissima* Petrulevičius et al., 2010	Neocomian, Lebanese amber	Petrulevičius et al. 2010
*Spinoberothamickaelacrai* Nel et al., 2005	Neocomian, Lebanese amber	Nel et al. 2005a
*Alboberothapetrulevicii* Nel et al., 2005	late Albian, Charentese amber (France)	Nel et al. 2005a
*Creagroparaberothagroehni* Makarkin, 2015	earliest Cenomanian, Burmese amber	Makarkin 2015a
*Eorhachiberothaburmitica* Engel, 2004	earliest Cenomanian, Burmese amber	Engel 2004
Paraberothinae sp.: Engel, 2004	earliest Cenomanian, Burmese amber	Engel 2004
* Micromantispa cristata * Shi et al., 2015	earliest Cenomanian, Burmese amber	Shi et al. 2015
*Scoloberothanecatrix* Engel & Grimaldi, 2008	earliest Cenomanian, Burmese amber	Engel and Grimaldi 2008
*Retinoberothastuermeri* Schlüter, 1978	early Cenomanian, Bezonnais amber (France)	Schlüter 1978
*Rhachibermissaphenax* Engel & Grimaldi, 2008	Turonian, New Jersey amber	Engel and Grimaldi 2008
*Rhachibermissasplendida* Grimaldi, 2000	Turonian, New Jersey amber	Grimaldi 2000
*Kujiberothateruyukii* gen. et sp. n.	middle Santonian, Kuji amber	this study
*Albertoberothaleuckorum* McKellar & Engel, 2009	Campanian, Canadian amber	McKellar and Engel 2009
** Rhachiberothinae **		
*Whalferavenatrix* (Whalley, 1983)	mid-Eocene, “British” amber*	Whalley 1983; Engel 2004
*Whalferawiszniewskii* Makarkin & Kupryjanowicz, 2010	mid-Eocene, Baltic amber	Makarkin and Kupryjanowicz 2010
**subfamily *incertae sedis***		
*Oiseacelinea* (Nel et al., 2005)	earliest Eocene, Oise amber	Nel et al. 2005a, b

*This amber is considered contemporarily with Baltic amber (Jarzembowski 1999).

## Materials and methods

**Figure 1.**
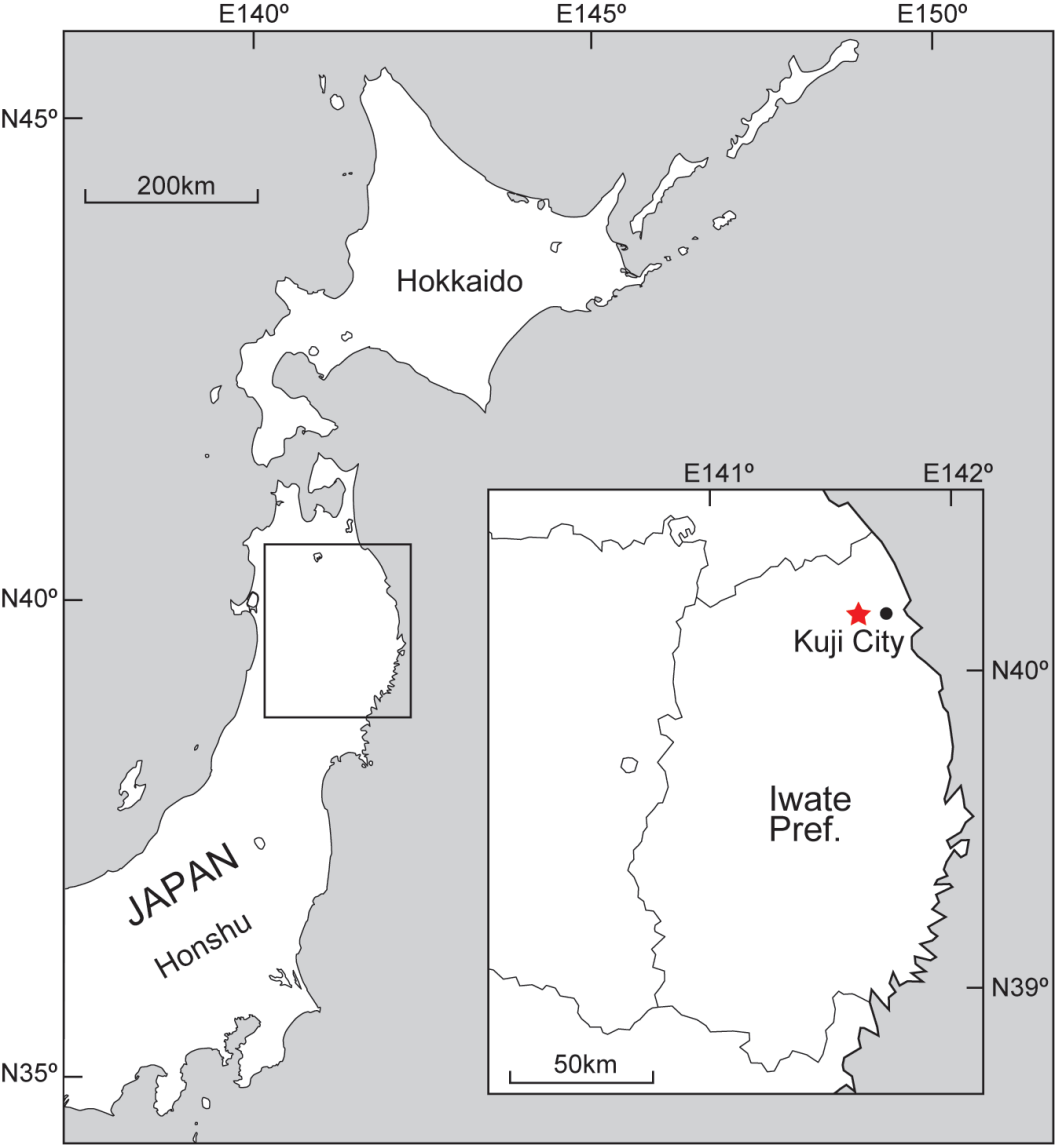
Map of the amber locality in Kuji City, Iwate Pref., northeastern Japan.

The specimen described in this study was found in the Kuji City, Iwate Prefecture, northeastern Japan (Fig. 1). The Kuji amber-bearing deposits are from the Upper Cretaceous Tamagawa Formation of the Kuji Group, the age of Kuji amber matrix from this locality has been estimated to be 83–90 Ma (Umetsu and Kurita 2007; Katagiri et al. 2013; Uno et al. 2018). Recently it was indicated that the age of Kuji amber matrix is dated to the middle Santonian, ca. 85.9 ± 0.7 Ma based on a U–Pb radiometric dating of zircon crystals of the volcaniclastic matrix (Arimoto et al. 2018). Kuji amber is the richest source of amber in Japan and it contains many paleontologically important fossils such as relatively abundant insects and a moss (e.g., Kawakami et al. 1994; Fursov et al. 2002; Katagiri et al. 2013).

**Figure 2.**
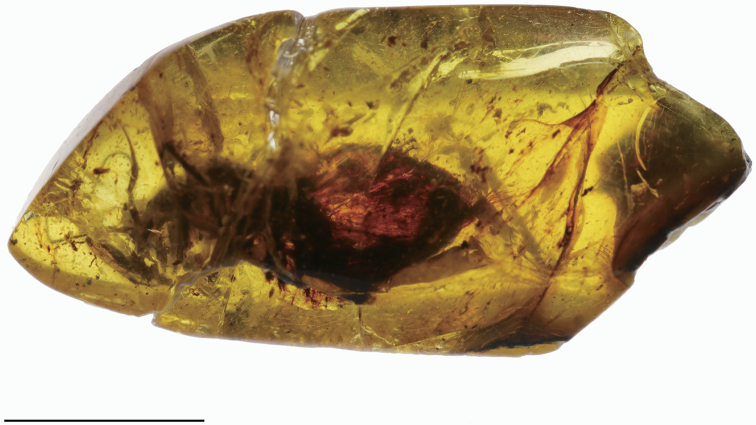
*Kujiberothateruyukii* gen. et sp. n., holotype. Overview of the whole inclusion in amber. Scale bar: 5.0 mm.

The specimen is embedded in a piece of elongated oval amber (18.6 × 8.6 × 4.7 mm) with some bubbles, debris, and deep cracks, covered with opaque substance and therefore only partly visible (Fig. 2). The holotype is housed in the Kuji Amber Museum, Kuji City, Iwate Prefecture, Japan.

We observed the specimen using a stereomicroscope SMZ745T and SMZ800 (Nikon corporation, Tokyo, Japan). The photographic data of the specimen was taken with the systems: Canon EOS 80D (Canon Inc., Tokyo, Japan) with EF–S 60mm F2.8 Macro USM (Canon Inc., Tokyo, Japan) plus Kenko Extension Tubes (KenkoTokina Co., Tokyo, Japan). Line drawings were prepared by using Adobe Photoshop CC 2018 and Adobe Illustrator CC 2018.

The terminology of wing venations generally follows Kukalová-Peck and Lawrence (2004) as interpreted by Yang et al. (2012).

## Systematic paleontology

### Order Neuroptera Linnaeus, 1758

#### Superfamily Mantispoidea Leach, 1815

##### Family Rhachiberothidae Tjeder, 1959

###### Subfamily Paraberothinae Nel et al., 2005

####### Genus Kujiberotha gen. n.

Taxon classificationAnimaliaNeuropteraRhachiberothidae

http://zoobank.org/D6F5C38C-7080-40E5-A8B6-6FBC748B309A

######## Type species.

*Kujiberothateruyukii* sp. n.

######## Etymology.

The new genus name is a combination of Kuji City (type locality of this specimen) and the generic name *Berotha*. Gender feminine.

######## Diagnosis.

Antennae moniliform, with at least 50 flagellomeres; forelegs raptorial, profemur long (ca. 1.9 mm), protibia covered with dense fine setae becoming slightly longer towards distal on dorsal edge, together with at least six short spines on ventral edge, probasitarsus with nine small spine-like setae on external ventral ridge; wings with fine setae densely on surface of each vein.

######## Differential diagnosis.

*Kujiberotha* gen. n. can be distinguished from the six paraberothine genera (*Paraberotha*, *Raptorapax*, *Creagroparaberotha*, *Eorhachiberotha*, *Rhachibermissa*, and *Albertoberotha*) by having much larger number of the flagellomeres of the antenna (*Kujiberotha* has over 50 antennal flagellomeres, while these genera have only 20–32 ones). From *Alboberotha* and *Micromantispa*, our new genus can be separated by having greater number of the spine-like setae on the probasitarsus (*Kujiberotha* has 9 setae on the probasitarsus, but there are only two such setae in *Alboberotha* and *Micromantispa*). *Kujiberotha* can be further discriminated from *Scoloberotha*, *Spinoberotha*, and *Chimerhachiberotha* based on the numbers of spines on the protibia (*Kujiberotha* has at least six spines, whereas *Scoloberotha* has only three; *Spinoberotha* has numerous sharp spines on the inner edge disposed in two rows; and, those of *Chimerhachiberotha* are comprised of numerous short setae). Furthermore, the probasitarsus of *Kujiberotha* is not distinctly elongated, while that of *Scoloberotha* is markedly elongated, even longer than the combined length of succeeding tarsomeres. Finally, *Kujiberotha* can be separated from *Retinoberotha* by the structure of the profemora. Namely, *Kujiberotha* has at least six long spines and numerous short spines on the ventral edge of the profemora; however, *Retinoberotha* alternatively has seven short, thin spines or fine setae on the inner lateral edge and they are restricted to the median area of the protibia (Schlüter 1978: fig. 37).

######## Systematic placement.

When this fossil was originally excavated in 2006 by Mr Kazuhisa Sasaki (the former director of the Kuji Amber Museum), it was identified as a member of the order Mantodea and this assignment has been believed to be correct until our study. In a recent summary of the fossil records of Mantodea (Delclòs et al. 2016), this undescribed fossil was placed as “Family *incertae sedis*” within Mantodea. However, we determined this fossil to be a thorny lacewing (Rhachiberothidae: Paraberothinae) based on the following morphological character states: antennae moniliform (filiform in Mantodea, except some taxa of Coptopterygidae, Empusidae, Hymenopodidae, Mantidae, Stenophyllidae, and Toxoderidae); probasitarsus with its external ventral ridge bearing several small spines and one long spine (Mantodea has a slenderer basal segment of the tarsus, lacking such spines); and simple wing venation (Mantodea usually has many crossveins). It is well known that Rhachiberothidae has a clearly raptorial-shaped foreleg, therefore this family can be easily distinguished from Berothidae (except Mesithoninae) (Aspöck and Mansell 1994). The synapomorphy of Paraberothinae is the presence of at least two spines on the inner edge of the protibia (usually with numerous spines; Nel et al. 2005a; Makarkin 2015a). However, there is no report for the presence of these protibial spines from all fossil and extant species of Mantispoidea (except Paraberothinae; uncertain in Mesoberothidae): namely, Rhachiberothinae, *Oisea*, Berothidae (including Mesithoninae), and Mantispidae (Aspöck and Aspöck 1997; Makarkin and Kupryjanowicz 2010; Makarkin 2015a, b). It is therefore noteworthy that *Kujiberotha* gen. n. has at least six spines on the inner edge of the protibia. This character alone supports the placement of *Kujiberotha* gen. n. within Paraberothinae.

####### Kujiberotha teruyukii sp. n.

Taxon classificationAnimaliaNeuropteraRhachiberothidae

http://zoobank.org/BF91E83A-6B50-4099-BEB2-3D7885D0D674

Figs 2 , 3 , 4

######## Material.

Holotype, incomplete specimen of adult, sex undetermined, deposited in the Kuji Amber Museum, Kuji City, Iwate Prefecture, Japan. This specimen is visible only in lateral view and many of the body parts are originally lost or difficult to observe.

######## Locality and horizon.

Kuji amber from the Kokujicho, Kuji City, Iwate Prefecture, northeastern Japan; Tamagawa Formation of the Kuji Group, middle Santonian (ca. 85.9 Ma; see Arimoto et al. 2018), Upper Cretaceous.

######## Etymology.

This remarkable mantispid-like insect is named in honor of the celebrated kabuki actor Mr. Teruyuki Kagawa. He is known for his love of mantises and is enormously popular with insect-loving children in Japan.

######## Diagnosis.

As for the genus (vide supra).

######## Description.

**Figure 3.**
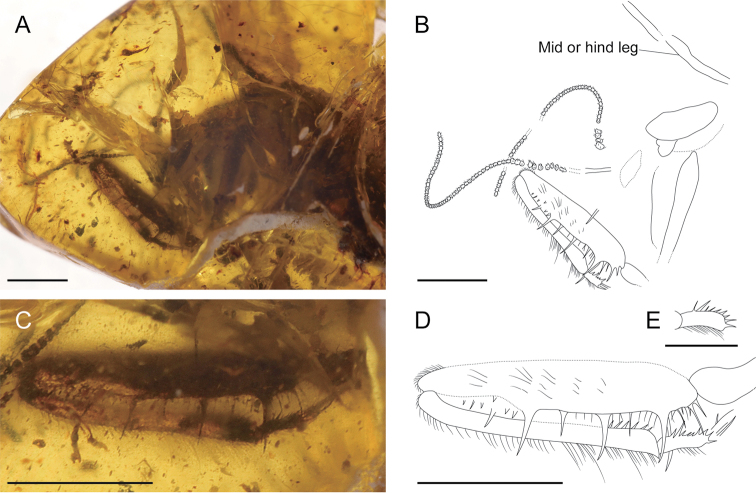
*Kujiberothateruyukii* gen. et sp. n., holotype. **A** photograph of anterior part **B** line drawing of anterior part **C** photograph of left foreleg **D** line drawing of left foreleg (outer lateral view) **E** line drawing of left probasitarsus (dorsal view). Scale bars: 1 mm (**A, B, C, D**); 0.5 mm (**E**).

**Figure 4.**
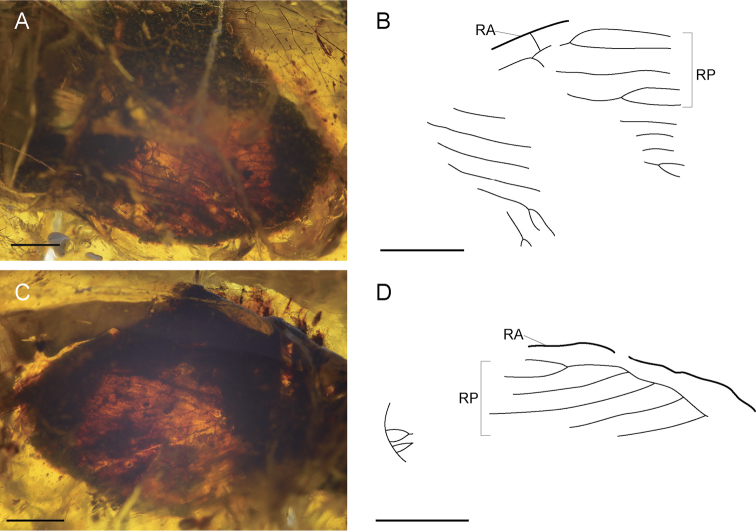
*Kujiberothateruyukii* gen. et sp. n., holotype. **A** photograph of forewing **B** line drawing of forewing **C** photograph of hindwing **D** line drawing of hindwing. Abbreviations: RA, anterior radius; RP, posterior sector. Scale bars: 1.0 mm.

*Head* entirely not clearly visible due to numerous cracks. Compound eyes partially visible. Antennae (Fig. 3A, B) moniliform, flagellum relatively long, composed of at least 50 flagellomeres, covered with fine setae on each segment. *Pronotum* elongate, visible only left lateral side, ca. 1.1 mm in length, with scattered setae on dorsal surface. Meso- and metathorax not visible. *Foreleg* (Fig. 3C, D) well preserved. Procoxa very long at least 1.7 mm, not broadened. Protrochanter elongate, slightly curved. Profemur exceedingly long ca. 1.9 mm, slightly broadened, dense fine setae on surface, several long spines and numerous short spines on ventral edge, only slightly curved towards distal. Protibia markedly long ca. 1.7 mm, slender, covered with dense fine setae becoming slightly longer towards distal on dorsal edge, six short spines visible bent towards distal on ventral edge. Protarsus partly preserved, probasitarsus elongate, dense fine setae on surface, with nine small spine-like setae on external ventral ridge (Fig. 3E) and single long curved spine distally. Other tarsomeres not well preserved. Mid- and hindlegs partly visible, slender, covered dense setae. *Abdomen* uniformly lost. *Wings* poorly preserved (Fig. 4), with dense fine setae on veins.

######## Remarks.

The profemur of *Kujiberothateruyukii* gen. et sp. n. is the longest among the Paraberothinae fossils found to date. The length of the profemur in this subfamily ranges from ca. 0.5 mm in *Spinoberothamickaelacrai* Nel et al., 2005 to ca. 1.14 mm in *Raptorapaxterribilissima* Petrulevičius et al., 2010. Meanwhile, that of *K.teruyukii* is notably longer, ca. 1.9 mm.

## Discussion

*Kujiberotha* gen. n. represents the first discovery of Rhachiberothidae from Japan and from East Asia, providing key insights into the past distribution and morphological diversity of thorny lacewings. In fact, the distribution of modern rhachiberothids is limited biogeographically to sub-Saharan Africa (Aspöck and Aspöck 1997). Although fossils of Rhachiberothidae have been reported from major amber deposits ranging from the Lower Cretaceous to the mid-Eocene, the localities of these fossil findings have previously been limited geographically (Table 1). This bias in fossil records is probably better explained by the locations of amber deposits than by the past distribution of rhachiberothids, based on the discovery of *Kujiberotha* gen. n. from Kuji amber. Our finding demonstrates that Paraberothinae was also distributed in the eastern part of Laurasia during the Cretaceous.

With 15 fossil genera, including *Kujiberotha* gen. n., Rhachiberothidae clearly possessed much greater generic diversity in the past than it does now. Indeed, the modern rhachiberothids are composed of only three genera. The discovery of *Kujiberotha* gen. n. adds further evidence for the potentially higher diversity of Rhachiberothidae during the Cretaceous. It seems reasonable to conclude that the remarkable morphological traits among the Cretaceous paraberothines were more diverse than those of other extinct and extant Rhachiberothidae (e.g., numerous long spines on the inner edge of the protibia, whereas all other rhachiberothids bear no spines). As mentioned above, the structures of the foreleg, particularly the presence of nine small spine-like setae on the external ventral ridge of the probasitarsus, have never before been reported from this family. Furthermore, the markedly large profemur of *Kujiberotha* gen. n. is quite unexpected and noteworthy. By contrast, some insects from Upper Cretaceous Burmese amber are miniaturized compared to modern taxa; for example, *Nicrophorus* and *Colon* beetles from this amber deposit are much smaller than their recent counterparts (Cai et al. 2014; Yamamoto and Takahashi 2018). Nonetheless, the true diversity of fossil Rhachiberothidae has not yet been adequately explored. Investigations into this subject should be conducted for amber from minor localities, such as Kuji, and for the Burmese amber due to its exceptionally abundant and diverse insect inclusions.

Kuji amber, with its long mining history, is the largest amber deposit in Japan. In spite of its importance, few studies have explored its insect inclusions (e.g., Kawakami et al. 1994; Fursov et al. 2002). More than 800 insect inclusions from Kuji amber still await formal descriptions (Kawakami et al. 1994). We hope that this paper will provide a foundation for studies of fossil insects in Kuji amber. Finally, we also expect that more fossil rhachiberothids will be discovered in the future, providing direct evidence of their distribution and morphological evolution to corroborate the hypothesis that thorny lacewings in the past were far more diverse than they are now.

## Supplementary Material

XML Treatment for Kujiberotha

XML Treatment for Kujiberotha teruyukii
